# Arthropod ectoparasites of synanthropic rodents in northern‐central Italy

**DOI:** 10.1111/mve.12818

**Published:** 2025-06-19

**Authors:** Filippo Maria Dini, Silvia Crucitti, Talita Bordoni, Roberta Galuppi

**Affiliations:** ^1^ Department of Veterinary Medical Sciences University of Bologna Bologna Italy

**Keywords:** ectoparasites, *Laelaps echidninus*, *Mus musculus*, Myobiidae, *Notoedres muris*, *Polyplax spinulosa*, *Rattus norvegicus*, *Rattus rattus*, Synanthropic rodents

## Abstract

Synanthropic rodents, due to their close contact with humans, have always been a concern because of their substantial impact on both the economy and public health. This study aims to investigate the presence of ectoparasites in rodents captured during pest control campaigns in various Italian provinces (Bologna, Forlì‐Cesena, Rimini, Ravenna, Arezzo) in northern‐central Italy. We examined a total of 140 carcasses of brown rats (*Rattus norvegicus* – 81 samples), black rats (*Rattus rattus* – 49 samples) and house mice (*Mus musculus* – 10 samples). Skin samples were collected, digested in sodium hydroxide and microscopically examined after the enrichment method. The results revealed the presence of *Polyplax spinulosa* lice in 44 subjects (31.4%), Myobiidae mites in 13 subjects (9.3%), *Laelaps echidninus* and *Notoedres muris* in 7 subjects respectively (5%) and mesostigmata mites in 3 (2.1%). A specimen of *Rattus norvegicus* was positive for the flea *Nosopsyllus fasciatus.* The conducted research has provided an overview of the arthropods present on the fur and skin of synanthropic mice and rats in the surveyed provinces. This study represents a baseline investigation, particularly considering the lack of nationwide and scarce European data.

## Graphical abstract

- The presence of ectoparasites in synanthropic rodents in various Italian provinces was investigated.
- Eighty‐one *Rattus norvegicus*, 49 *Rattus rattus* and 10 *Mus musculus* were examined
- *Polyplax spinulosa* lice, Myobiidae mites, *Laelaps echidninus* and *Notoedres muris* were mainly recovered.

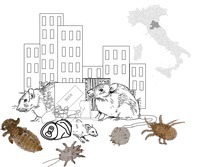

## INTRODUCTION

The term “synanthropic” refers to animal species that have developed close associations with environments influenced by human activity (Baumann, 2023; Hulme‐Beaman et al., 2016). During the period of sedentarization, when humans started to establish stable ecological niches, rodents were among the first animals attracted to these new, consistent source of food (Baumann, 2023). Synanthropic rodents such as the brown rat (*Rattus norvegicus*), black rat (*Rattus rattus*) and house mouse (*Mus musculus*) are listed among the 100 most invasive and harmful species globally, according to the IUCN. They are present worldwide, with the exception of the Antarctica, and occupy a broad range of ecological niches (Puckett et al., 2016). Their presence can significantly alter ecosystems, change vegetation patterns, and cause decline or extinction of native species, through direct predation and depletion of food resources (Gotti et al., 2022). In urban environments, the abundance of food and inadequate waste management create favourable conditions for these species, which are well known for destroying stored food (Feng & Himsworth, 2014) and damaging infrastructure (Schweinfurth, 2020). Moreover, their presence can be associated to the spread of communicable diseases that can affect both domestic animals and humans (Agrimi & Mantovani, 1996; De Sabato et al., 2024; Di Bartolo et al., 2024; Hornok et al., 2015; Meerburg et al., 2009), including parasitic infections (Dini, Caffara, et al., 2024; Dini, Mazzoni Tondi, & Galuppi, 2024; Magri et al., 2022), thereby posing an increasing threat to public health (Himsworth et al., 2014).

Among the parasites that affect rodents, ectoparasites also play a significant role. The complex relationship between rodent synanthropism and ectoparasites has been especially examined in the context of rat fleas (such as *Xenopsylla cheopis* and *Nosopsyllus fasciatus*) and their role in the transmission of *Yersinia pestis* (Bramanti et al., 2019; Eisen et al., 2015; Hinnebusch et al., 2017). However, fleas and other rodents' ectoparasites, including lice, ticks and various mite species, have also been implicated in the transmission of bacteria and viruses (Alghamdi, 2019; Gu et al., 2022; Gutiérrez et al., 2015; Herrera‐Mares et al., 2022). For instance, *N. fasciatus* can serve as a vector for trypanosomes and *Salmonella* spp. (Hamidi & Bueno‐Marí, 2021). The mite *Ornithonyssus bacoti* could be of public health concern, as it can occasionally infests carnivores, birds and humans (Mullen & O'Connor, 2019) and experimental studies have demonstrated its ability as a vector for *Rickettsia* spp., *Coxiella burnetii*, hantaviruses, *Borrelia* spp. and *Bartonella* spp. (D'Ovidio et al., 2018). The louse *Polyplax spinulosa* is a competent vector of *Rickettsia* spp., *Borrelia* spp. and *Bartonella* spp. It also plays a role in maintaining *Rickettsia typhi* and, although rarely, *Yersinia pestis* within rodent populations (Alonso et al., 2020; Reeves et al., 2006; Traub et al., 1978). The mite *Laelaps echidninus* is known to biologically transmit pathogens such as the causative agent of murine typhus among wild rodents. It has also been implicated in the transmission of the Junin virus, the etiological agent of Argentine haemorrhagic fever, a severe and potentially fatal disease primarily affecting agricultural workers in South America (Kolokoltsova et al., 2014). Additionally, mites belonging to the family Laelapidae are recognized as vectors of hantaviruses responsible for haemorrhagic fever with renal syndrome (HFRS) (Zhang et al., 2014).

Given these considerations, it is crucial to understand the distribution of ectoparasites in synanthropic rodents inhabiting anthropized areas. Researches on this topic are limited and fragmented in literature, with most studies focusing primarily on synanthropic rats (Alonso et al., 2020; Frye et al., 2015; Islam et al., 2021; Mlik et al., 2022; Soliman et al., 2001). In particular, very little is known about the ectoparasites of mice and rats in Italy. Only two studies have been published, both focused on Sicily, the largest island in the Mediterranean (Milazzo et al., 2003; Virga et al., 1993) with no data available from the mainland. In this study, we conducted a survey of the ectoparasites found on *Rattus rattus*, *Rattus norvegicus* and *Mus musculus* collected in urban and suburban areas of northern‐central Italy, where prior data are lacking.

## MATERIALS AND METHODS

**FIGURE 1:**
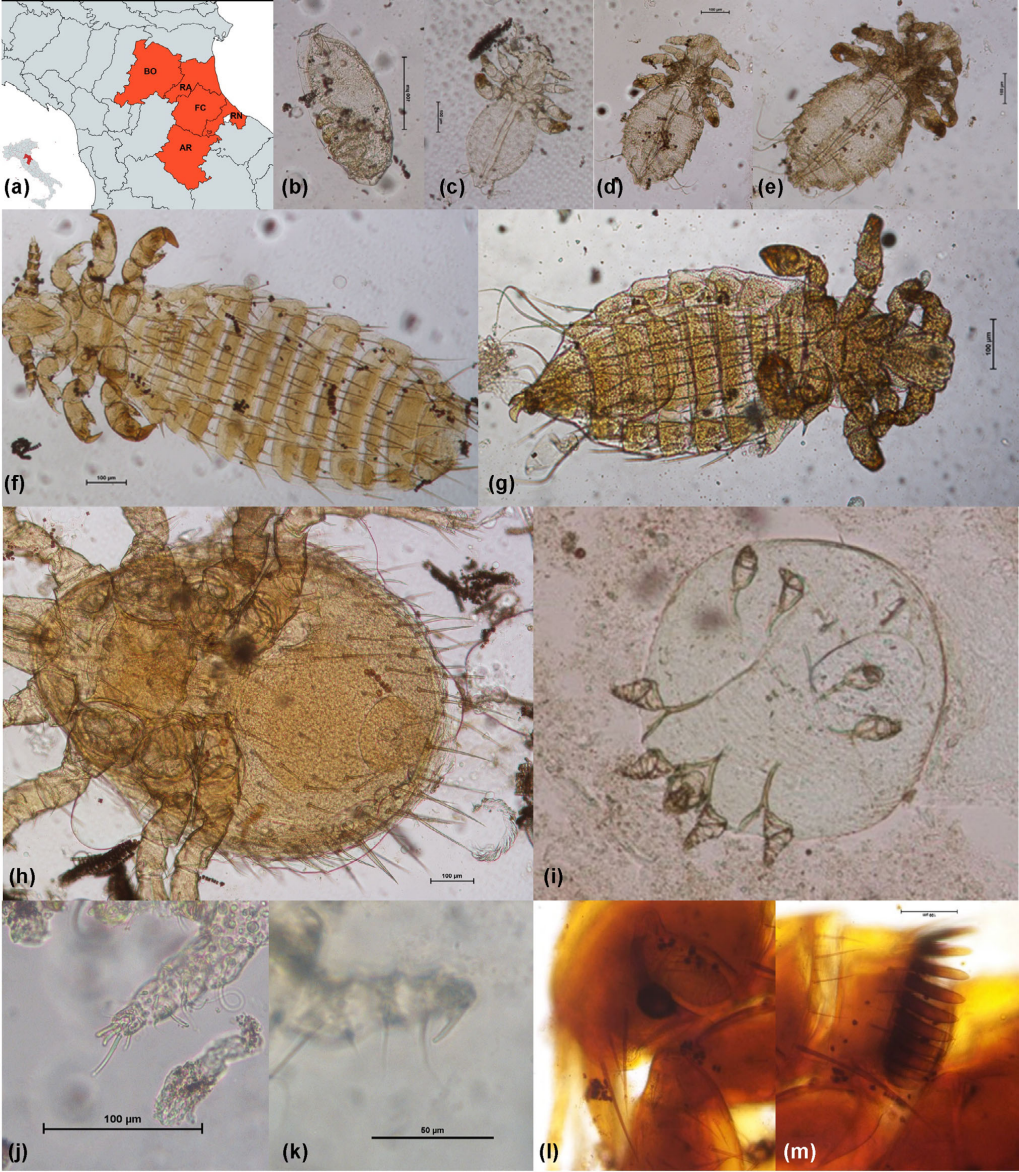
(a) Area of sampling, created with mapchart.net. Provinces: AR, Arezzo; BO, Bologna; FC, Forlì‐Cesena; RA, Ravenna; RN, Rimini; (b–m) *Polyplax spinulosa*: (b) egg, (c) first instar nymph, (d) second instar nymph, (e) third instar nymph, (f) adult female, (g) adult male; (h) *Laelaps echidninus*, female, ventral view; (i) *Notoedres muris*, female, ventral view; (j) *Radfordia ensifera*: paired and equal claws on the tarsus of the second pair of legs; (k) *Myobia murismusculi*: single claw on the tarsus of the second pair of legs; (l, m) *Nosopsyllus fasciatus*, anterior and posterior part of the head.

A total of 140 synanthropic rodents were examined, including 81 *R. norvegicus*, 49 *R. rattus* and 10 *M. musculus*, collected from the provinces of Bologna, Forlì‐Cesena, Ravenna, Rimini and Arezzo (Figure 1a). These specimens were obtained during rodents control campaigns. Specifically, the company For.B, based in Forlì and operating throughout the region, contributed carcasses from the provinces of Forlì‐Cesena, Rimini and Ravenna. For.B primarily uses snap traps and occasionally rodenticides in its control efforts. Additional samples were obtained from a rodent control campaign at the Zoosafari in Ravenna. In the provinces of Arezzo and Bologna, carcasses were provided by private individuals who had used snap traps during routine rodent control activities.

All samples were labelled according to their area of origin and stored at −20°C by field operators until they were transferred to the Department of Veterinary Medical Sciences (DIMEVET). Prior to necropsy, each carcass was recorded for species—identified by morphological characteristics (Iannino et al., 2023) ‐as well as weight, sex and place of origin. During necropsy, the skin of each rodent was visually inspected for macroscopically visible ectoparasites (Milazzo et al., 2003). In addition, a skin sample approximately 3 × 3 cm in size was collected using scissors from the dorsal region, specifically the sacral area. Each skin flap was placed in a Falcon tube and stored at −20°C until further analysis.

Each tube containing the skin sample was thawed by refrigerating overnight at 4°C. A 10% aqueous sodium hydroxide (NaOH) solution was then added to completely submerge the tissue, followed by incubation at 37°C for 6 hours. This digestion process facilitated the dissolution of the epidermis and hair. The resulting suspension was centrifuged, and the sediment was washed twice with distilled water by centrifugation. The processed sediment was then subjected to a flotation technique using a 1300 PS solution (Di Felice & Ferretti, 1962) and examined microscopically. The recovered ectoparasites were first preserved in 70% ethanol, then cleared with lactophenol for enhanced transparency. Identification was carried out using standard taxonomic keys (Baker & Wharton, 1952; Krantz, 1971; Pratt & Karp, 1953; Séguy, 1944).

Data on the examined animals and the identified ectoparasites were recorded in an Excel database. A descriptive analysis was performed to calculate the frequencies of ectoparasite recovered.

## RESULTS

A total of 140 rodent skins analysed, of which 56 (40%) tested positive for mites, lice or fleas (Table 1). No ticks were recovered in our sample set.

**TABLE 1:** Ectoparasites found in rodents from three provinces investigated: RA = Ravenna; FC = Forlì‐Cesena; BO = Bologna.

Species	Province of origin	n. examined	Positive	*Radfordia* sp.	*L. echidninus*	*N. Muris*	*Other mites*	*P. Spinulosa*	*N. Fasciatus*
*R. norvegicus*	RA	23	14 (60.9%)	5	2	6	0	9 [3]	1
	FC	33	15 (45.4%)	4	0	1	0	7 [5]	0
	BO	25	9 (36%)	1	4	0	1 *Mesostigmatae*	6	0
*R. rattus*	RA	18	4 (22.2%)	0	0	0	0	4	0
	FC	18	10 (5.6%)	1	1	0	2 *mesostigmata*	6 [2]	0
	BO	1	1 (100%)	0	0	0	0	[1]	0
*M. musculus*	RA	3	0	0	0	0	0	0	0
	FC	5	2 (40%)	1 (Myobia)	0	0	0	1	0
	BO	2	1 (50%)	1 (Myobia)	0	0	0	0	0

*Note*: In square brackets is the number of additional samples with only lice eggs. The black rats examined from the provinces of Rimini (1) and Arezzo (11) are not reported as no ectoparasites were found.

Among the 81 *Rattus norvegicus* examined, there were 48 males, 28 females and 5 individuals of unknown sex. Of the total specimens analysed, 46.9% tested positive for one or more ectoparasites.

Lice were detected in 37% of the individuals; in 8 cases, only eggs were found, whereas 27.16% of rats harboured various developmental stages of *Polyplax spinulosa* (Figure 1b–g). Mites were identified in 25.9% of specimens: *Radfordia ensifera* (Figure 1j) was morphologically identified in 10 cases, *Laelaps echidninus* (Figure 1h) was present in 6.4% of samples and *Notoedres muris* (Figure 1i) was observed in 8.64% of individuals. Additionally, degenerated mesostigmatid mites were found in one sample.

Furthermore, a single specimen (1.2%) harboured a flea of the species *Nosopsyllus fasciatus* (Figure 1l,m), in association with lice eggs, *R. ensifera* and *N. muris*. Co‐infestation of lice with one or more mite species was detected in 12 individuals.

In some instances, the presence of arthropods likely attributable to environmental contamination was observed. Specifically, one sample contained two distinct deutonymphal (hypopal) forms, presumably belonging to the families Glyciphagidae and Saproglyphidae. In addition, insect fragments from the genus *Liposcelis* were identified in two samples. Lastly, post‐mortem contamination by dipteran eggs and larvae was detected in four individuals.

Among the 49 *Rattus rattus* specimens analysed (18 females and 31 males), 15 individuals (30.6%) tested positive for one or more arthropod‐related elements.

Lice infestation was detected in 20.4% of the specimens, with *P. spinulosa* identified in 10 individuals, while only lice eggs were found in three cases. Four black rats (8.2%), all from the province of Forlì‐Cesena, tested positive for mites: *Radfordia ensifera* was observed in one case (2%), *Laelaps echidninus* was detected in another (2%) and in two samples, degenerated mesostigmatid mites were identified in association with *Polyplax spinulosa*.

No ectoparasites were detected in the black rats examined from the provinces of Rimini (*n* = 1) and Arezzo (*n* = 11); therefore, these samples are not included in Table 1. Additionally, environmental contaminants, including altered mites, were observed in six samples. Furthermore, insect specimens identified as *Liposcelis* sp. were found in two samples, and post‐mortem contamination by dipteran larvae was noted in one case.

Among the 10 *Mus musculus* specimens analysed (9 males and 1 female), only 3 individuals (all adult males) tested positive for ectoparasites. *Polyplax spinulosa* was identified in one specimen (10%), while *Myobia murismusculi* (Figure 1k) was detected in two specimens (20%).

## DISCUSSION

In this survey conducted across five provinces of northern‐central Italy, 56 out of 140 rodents (40%) tested positive for ectoparasites. To our knowledge, studies on rodent ectoparasites in Italy are limited to those conducted in Sicily (Milazzo et al., 2003; Virga et al., 1993). Specifically, Milazzo et al. (2003) reported the presence of only fleas and ticks in *Rattus rattus* and *Mus musculus*. In contrast, our study found no ticks and detected only a single *Rattus norvegicus* specimen positive for fleas, specifically *Nosopsyllus fasciatus*. This species was also reported in the two previous studies from Sicily and is considered a cosmopolitan ectoparasite that infests various rodent hosts.

Despite its global distribution, and contrary to expectations, mesostigmata mites of the species *Ornithonyssus bacoti* ‐commonly known as the tropical rat mite and occasionally reported by Virga et al. (1993) in a single *R. rattus* in Sicily – were not detected in our survey. However, altered mesostigmata mites were found in one *Rattus norvegicus* and two *Rattus rattus* specimens, without definitive identification. *Ornithonyssus bacoti* is known for its low species specificity, and it has been frequently reported in southern Italy, particularly in various exotic companion animals (D'Ovidio et al., 2018). This mite is also commonly described in synanthropic *R. norvegicus* (Alonso et al., 2020; Frye et al., 2015) and *R. rattus* (Mlik et al., 2022) from other continents.

In our study, the low prevalence of flea‐positive rodents and the absence of ticks and *O. bacoti* may be attributed to the method of sample collection. The rodents were obtained through pest control operations involving rodenticides or snap traps, and carcasses were not retrieved daily by pest control workers, as suggested by the presence of dipteran larvae in some specimens. *Ornithonyssus bacoti* is an intermittent blood feeder, and typically, only individuals in the bloodsucking stage are found on the host (Flynn, 1973). When rats are killed or abandon their nests or runways, the mites are left behind (Mullen & O'Connor, 2019). Similarly, fleas and ticks are known to leave the hosts after death; in particular, ticks may detach and seek a new host to complete their blood meal (Sonenshine, 2009). Furthermore, investigating the type of rodenticide used during control effort could be informative, as certain formulations may contribute to the mortality or detachment of ectoparasites (Hinds et al., 2021).

Among the 140 rodents examined, 44 individuals (31.4%) tested positive for sucking lice, with infestations primarily observed in *Rattus* spp. All lice were identified as *Polyplax spinulosa*. A similar prevalence (37.7%) was reported by Virga et al. (1993) in *Rattus* spp. from Sicily. According to recent studies, *P. spinulosa* is the most common species of sucking louse found on *Rattus* spp. in the Middle East and Algeria (Islam et al., 2021; Mlik et al., 2022). It has been detected in up to 73.9% of *Rattus norvegicus* specimens in New York City (Frye et al., 2015) and in 38% of rodents from rural areas of Buenos Aires (Alonso et al., 2020). Although sucking lice are typically highly host‐specific, *P. spinulosa* has been reported in nine species of rats (Wang et al., 2020) as well as in domestic mice (*Mus musculus*) (Abdel‐Rahman et al., 2020; Malla & Khurshid & Saida, 2022). In our study, one *M. musculus* specimen was also found to be infested.

Fur mites of the family Myobiidae are obligate ectoparasites of rodents, bats, insectivores and certain marsupials. Over time, they have adapted to life within the fur of their mammalian hosts, anchoring themselves to hair shafts using specialized forelegs. Typically, *Radfordia ensifera* parasitizes *Rattus* spp., while *Myobia murismusculi* is most commonly found on house mice (*Mus musculus*) (Mullen & O'Connor, 2019). In the present study, *R. ensifera* was primarily detected in *Rattus norvegicus* (10 out of 81 specimens, 12.3%) and in a single *Rattus rattus* individual (1/49, 2%). *Myobia murismusculi* was observed in 2 out of 10 *M. musculus* specimens. These fur mites have not been reported in synanthropic rodents from Sicily (Virga et al., 1993), Buenos Aires (Alonso et al., 2020), New York (Frye et al., 2015) or Algeria (Mlik et al., 2022), although they have been documented in Croatia (Stojcevic et al., 2004) and are frequently reported in laboratory rats and mice (Flynn, 1973).

Necropsy of seven *R. norvegicus* specimens, from which skin samples were collected, revealed crusty lesions and erosions on the nose, tail and ears. Microscopic examination confirmed the presence of *Notoedres muris*, a mite responsible for notoedric mange, a condition frequently observed in laboratory *Rattus* spp., though it is not known to infest humans (Mullen & O'Connor, 2019). While *N. muris* was not reported in the aforementioned studies on synanthropic rodents, it was previously detected in a localized area of Vancouver, Canada, through histological analysis of rodent skin samples (Anholt et al., 2014).

Many of the previously cited studies primarily relied on ectoparasite collection through hair clipping and macroscopic examination of captured and recently euthanized animals. While the post‐mortem collection of rodents in this study limited the detection of *O. bacoti*, fleas and ticks, as mentioned earlier, the digestion of skin samples in sodium hydroxide, followed by sedimentation and flotation, likely increased the sensitivity of our method particularly for detecting Myobiidae mites (which firmly grip the fur) and *N. muris* (which resides within the keratinized layers of the skin).

In seven samples (six *R. norvegicus* and one *R. rattus*), *Laelaps echidninus* was detected. This ectoparasite has also been reported in Sicily (Milazzo et al., 2003; Virga et al., 1993), Buenos Aires (Alonso et al., 2020) and New York (Frye et al., 2015). According to Jakeman (1961), the primary hosts of *L. echidninus* are predominantly *Rattus* spp.

The presence of hypopi and adult dust mites (Glyciphagidae and Saproglyphidae) on the fur of synanthropic rodents is not unexpected, as they are commonly found in household environments. Similarly, *Liposcelis* spp., Psocoptera insects known as booklice due to their preference for old books, are also occasionally encountered. While not parasitic, these insects often coexist with mammals and birds, residing in their nests, dens, fur or feathers and feeding on fungi or organic material. In this context, the examined rodents essentially act as carriers of these arthropods, playing a phoretic role in their dispersal. Although these arthropods are generally not harmful, they can cause occasional dermatitis in humans and allergic reactions.

## CONCLUSION

This survey of synanthropic rodents in northern‐central Italy reveals that *Polyplax spinulosa*, the sucking louse, is one of the most prevalent ectoparasites, consistent with its global distribution in rats and mice. The diversity and distribution of ectoparasites in rodents are influenced by a combination of host, parasite and environmental factors, including human activity. Notably, most of the samples positive for *Notoedres muris* were from a zoo safari, suggesting potential exposure to various animal species. Despite challenges such as sample conditions that likely hindered the detection of ticks, the skin dissolution and enrichment techniques allowed for the identification of Myobiidae and *N. muris*, which are rarely reported unless specifically targeted. This study, though limited, provides a valuable snapshot of the ectoparasites affecting synanthropic rodents in the investigated area, filling a gap in previous research.

## AUTHOR CONTRIBUTIONS

**Filippo Maria Dini:** Investigation; methodology; writing – original draft; writing – review and editing. **Silvia Crucitti:** Investigation; writing – original draft; writing – review and editing. **Talita Bordoni:** Investigation; writing – review and editing. **Roberta Galuppi:** Investigation; visualization; writing – original draft; writing – review and editing.

## CONFLICT OF INTEREST STATEMENT

The authors declare that they have no competing interests.

## ETHICS APPROVAL STATEMENT

The research described in this manuscript does not fall within Directive 63/2010 of the European Parliament and of the Council on the protection of animals used for scientific purposes (transposed into Italian law by Legislative Decree 26/2014) and thus does not require any authorization from the national competent Authorities.

## Data Availability

The data that support the findings of this study are openly available in AMS Acta Alma Mater Studiorum – University of Bologna at https://amsacta.unibo.it/. https://amsacta.unibo.it/id/eprint/8341/ [Correction added on 1 July 2025 after first online publication: The Data Availability Statement has been updated.]
